# Modern Magicians and Mediomaniacs

**Published:** 1860-04-01

**Authors:** 


### Art. X.—MODERN MAGICIANS AND MEDIOMANIACS.

It is a huge mistake to imagine that the magicians of our own days are in any respect inferior to those of an earlier date. If there be any so utterly ignorant that they had believed that magicians were, at least so far as Europe was concerned, peculiarities of times long past, to them we would especially commend this paper, which is neither more nor less than a contributory waif to the psychological history of our own time. It may be that the magicians of a former and remote period exercised a greater influence over the minds of the people at large than those who exist now. Not, however, because the latter have drunk less deeply from the fountain of the occult sciences, or that they wield less potent powers, or have less faith in them, but because they have not escaped from the mollifying influence of an advancing civilization, and have thus become less fitted to deal with, and perhaps more heedless of, the increasing perversity and unbelief of the vulgar herd (although, by the way, the ingestive capacity of this in the matter of mystical odds and ends is by no means mediocre, witness for example, spirit-rapping). The exercitations of the occult brotherhood are, notwithstanding, of far too great an interest as illustrations of the psychical eccentricities of the present epoch to be lost sight of, and we propose to cull a few examples to this end from the latest specimen which has come to light, to wit, the *History of Magic*,* by Eliphas Lévi. This name is a pseudonym, and it may be translated, *associated in God's work.* The author is, we believe, a priest; consequently

* *Histoire de la Magie; avec une Exposition claire et précise de ses Procédés, de ses Rites, et de ses Mystères.* Par Eliphas Lévi, auteur de *Dogma et Ritucl de la haute Magie.* Paris. 1860.

the pseudonym may be looked upon as a double hit, applying on the one hand to his actual duties, on the other to his labours in conjunction with others in the cause of magic. He has inscribed on the title-page of his book, Khunrath's definition of a great work —*Opus Hierarchicum et Catholicum*.

The construction of the work is one befitting its subject, as the framework is built up out of the science itself of which the book treats. It must be premised that the present work is the second instalment of a complete course of magic, to be terminated in three parts. The first portion, published in 1856, treats of the *Dogmas and Ritual of High Magic;* the second portion is the one now under consideration; and the third, to be entitled the *Key to the Great Mysteries*, will be forthcoming sooner or later.

This threefold division of our author's lucubrations has been determined, it would appear, by magical rule and precept. The discovery of the great mysteries of magic rests entirely, he tells us, upon the signification attached to numbers by the ancient hierophants. THREE was regarded by them as the generative number, and in teaching any doctrine they first considered the theory, next the realization, then the adaptation to every possible usage. "Thus," writes the so-called Eliphas Lévi, "are formed dogmas, whether philosophical or religious. For example, the dogmatic synthesis of Christianity, inherited from the Magi, imposes upon our faith three Persons in one God, and three mysteries in the universal religion."—(p. 5.)

Our author, therefore, follows in the primary divisions of his work the ternary plan laid down by the Cabbalah, "that is to say, the foremost tradition of the occult sciences." Further, he is guided in the subdivisions of his writings also by the mysteries of numbers. Thus the *Dogma* and *Ritual* are each divided into twenty-two chapters marked by the twenty-two letters of the Hebrew alphabet. He places at the head of each chapter the letter which belongs to it, adding "the Latin words which, according to the best authors, indicate the signification of the hieroglyphic." Thus he heads the first chapter in this manner :—

I א A

LE RECIPIENDAIRE,

*Disciplina,*

*Ensoph,*

*Keter.*

We are instructed that the letter *aleph*, of which the equivalent in Latin and French is A, and the numerical value 1, signifies the candidate, the man about to be initiated, the dexterous individual (the card-juggler—le bateleur du tarot). It signifies the dogmatic syllepsis (*disciplina*); Being (l'être) in its primary and general conception (Ensoph); finally, the first and obscure idea of the divinity expressed by *Keter* (the crown) in the cabbalistic theology. "The chapter is the development of the title, and the title contains hieroglyphically all the chapter. The entire book is composed according to this combination."

The *History of Magic*, the book which immediately concerns us, recounts and explains the realizations of the science in the course of ages, and it is arranged according to the number *seven* this being the representative of the week of Creation, and of the divine consummation.

The *Key to the Great Mysteries* is to be based upon the number *four*, this it appears being that of the enigmatical forms of the Sphynx and of the elementary manifestations. In this book, we are told, will be fully explained the enigma of the Sphynx, and will also be presented the key to those things which have been hidden since the beginning of the world.

Let us then learn from this new Œdipus somewhat of the wondrous science by which these marvels may be wrought. It is evident that he may justly exclaim with Mephistopheles, when he seated himself among the Sphynxes—

> "How easily I make myself at home!
> I understand each spirit's tongue around me."

MAGIC, then, "is the science of the ancient Magi; and the Christian religion, which has silenced the lying oracles and put an end to the prestiges of false gods, itself reveres those Magi who came from the East, guided by a star, to adore the Saviour of the world in his cradle." Tradition has given to these Magi the titles of *kings*, "because (mark the reasons) the initiation into magic constitutes a true royalty, and because the great art of the Magi is termed by all its adepts the *royal art*, or the *holy kingdom, sanctum regnum*."—(p. 2.)

Magic unites in one and the same science all that is certain in philosophy and all that is infallible and eternal in religion. It links together perfectly and incontestably faith and reason, science and belief, authority and freedom—forms which at first seem so opposed. "It gives to the human mind an instrument of philosophical and religious certitude exact as mathematics, and explains the infallibility of mathematics themselves."—(p. 2.)

We shall quickly have an opportunity of seeing this wonderful instrument tested.

Thus, then, we learn, that there is an absolute in the matters which concern intelligence and faith. Supreme reason does not suffer the gleams of the human understanding to be blown about by the puffs of every idle wind. There does exist an incontestable truth, and by means of this truth, once seized upon, men who take it for their guide can give to their volition "a sovereign puissance which will render them masters of all things inferior to them, and of all wandering spirits, that is to say, arbiters and kings of the world."—(p. 3.)

Well may we ask, guided by our author, if it be thus, how comes it that this science is unknown? How can we imagine that so glorious a sun exists in a heaven that we know to be most obscure? How? Alas! that the brimming cup should be dashed to the ground the moment we attempt to raise it to our lips. But so it is. The "lofty science," we are told, "has always been known, but solely by those understandings which belong to the *élite*, and who have comprehended the necessity of being silent and of waiting. If an able surgeon were, in the middle of the night, to give sight to one who was blind, how could he comprehend before the morning had dawned the existence and nature of the sun?"

Again, we are taught that this science can never be vulgarized,

"— Because it is hierarchical, and because anarchy alone flatters the prejudices of the multitude. Absolute truths are not necessary for the masses, otherwise progress would be arrested, and life would cease in humanity, the hither-and-thither of contrary ideas, the shock of opinions, the passions of the world, determined always by the dreams of the moment, are necessary to the intellectual increase of nations. (How charming is this candour of the *élite!* *They* possess bread, but *we* must rest content with stones.) The multitudes do not lack feeling, and it is for this reason that they abandon so willingly the chair of the professor in order to hasten to the trestle of the charlatan. Men even who are supposed to occupy themselves especially with philosophy, resemble almost always children who amuse themselves with enigmas, and who invariably send away first those who may chance to know the solution, lest these should put an end to the game by destroying all the interest which appertains to the embarrassment excited by the questions."—(p. 6.)

It is always thus with these men of transcendental knowledge. Like Ralpho's "New Light"—

"'Tis a dark lanthorn of the spirit
Which none see by but those that bear it.

. . . . . . . . .

Although they promise strange and great
Discoveries of things far fet,
They are but idle dreams and fancies,
And savour strongly of the ganzas.
Tell me but what's the natural cause
Why on a sign no painter draws
The full moon ever, but the half?
Resolve that with your Jacob's staff;

Or why the wolves raise hubbub at her,
And dogs howl when she shines in water?
And I shall freely give my vote,
You may know something more remote."

It is well, however, that we should seek to know somewhat more precise respecting the potency of magical science; and seeking, we find, that if we conform ourselves to the rules of the eternal force (whatever that may be), man can assimilate himself to the creative power and become creator and conservator like unto it. God—our author is pre-eminently religious after a fashion, and he has a habit of tagging a Scriptural illustration or quotation to the opinions he emits—God, it would seem, has not restricted to a limited number the steps of Jacob's luminous ladder. Whatever exists in nature inferior to man is submitted to him, and to him belongs the faculty of increasing indefinitely his dominion in always ascending:—

"Thus the length and even the perpetuity of life, the atmosphere and its storms, the earth and its metallic veins, light and its prodigious mirages, night and its dreams, death and its phantoms, all these obey the royal sceptre of the Magi, the pastoral staff of Jacob, and the terrible rod of Moses. The adept becomes king of the elements, a metamorphoser of metals, arbiter of visions, director of oracles, and master of life, according to the mathematical order of nature, and conformably to the will of the supreme intelligence. There is magic in all its glory! But who will dare, in our age, to give faith to our words? Those who desire loyally to study and frankly to learn; because we shall no longer hide the truth under the veil of parables or of hieroglyphical signs, the time has come when all should be made known, and we propose to do it."—(p. 9.)

If the right apprehension of this knowledge is, however, reserved for the elect, it is to be feared that we shall profit as little by an explanation of the modes in which it is to be attained, as Jessica did from Lorenzo's disquisition on the music of the spheres:—

" For while this muddy vesture of decay
Doth grossly close us in we cannot hear it."

M. Lévi's account of the power possessed by the modern magician, magniloquent though it may be, scarcely satisfies us. It certainly does not fail in assigning to magical science a sufficiency of power; but we still want something a little more definite; we want, in short, a few subsidiary details. We shall turn, therefore, for a few moments to M. Cahagnet's confession of faith, he being a contemporary of M. Lévi, and one who reposes trust in *animal magnetism*. But both he and M. Lévi mean the same thing, and go over pretty much the same ground.

M. Lévi is, however, much the more honest and praiseworthy writer of the two, for he frankly adopts magic as a science, while M. Cahagnet seeks to smuggle into favour the phenomena of magic under the guise of a pseudo-science.

M. Cahagnet's confession is given in the form of question and answer, and lest we should be thought guilty of gross exaggeration, we must transcribe it literally:—

"Can we produce a cataleptic state by the action of human magnetism?—Yes.

"Can we subtract or triple the powers of a magnetic subject submitted to our action?—Yes.

"Can we produce upon this subject those effects of attraction, which every magnetizer assures us have been produced, not only upon animated beings, but upon inanimate bodies?—Yes.

"Can we by means of this attraction occasion the suspension of natural bodies?—Yes.

"Can certain subjects in the magnetic state execute gymnastic movements, as well as movements inadmissible by the laws of anatomy?—Yes.

"Can an individual in this state attain a taller growth than natural?—Yes.

"Can he walk upon *points d'appui* contrary to the constitution of his being, and to the laws of equilibrium?—Yes.

"Can he produce upon his person boundless local and general inflammations?—Yes.

"Can he in this state see, the eyes being closed, either by the neck, the stomach, or the fingers, at incommensurable distances, and hear what is said there?—Yes.

"Can the so-called spirit separated from matter, have material affinities?—Yes.

"Can the *lucid* speak many languages which are unknown to him, as well as acquire a knowledge of science of which he was always ignorant?—Yes.

"Can he in this state defy the action of fire and poisons?—Yes.

"Can he communicate with the dead, speak to them, and ascertain from them useful things?—Yes.

"Can he, in his turn, fascinate his magnetizer by rendering himself, or such objects as he wishes, invisible at his will?—Yes.

"Can the magnetizer possess his subject by sounds which he makes him hear from a distance, work upon him the effects of attraction also at a distance, produce apparitions of beings or fantastic objects, and force him thus to do things against his repose, morality, and honour?—Yes.

"Can the magnetizer in this manner render any one idiotic or mad, or even kill, without any *traces being visible*, the victim submitted to his action?—Yes.

"Can he induce any malady whatsoever, or destroy the use of a limb?—Yes.

"Can he give blows from very great distances?—Yes.

"Can he lead persons astray, cause them to leap ditches, or to drink

*au chalumeau*,* create obstacles in straight roads, and occasion the appearance as it were of robbers or ferocious animals ?—Yes.

"Can he throw stones into places afar off, without being seen, and bewitch lands, gardens, beasts, and men, as all books on sorcery aver ?—Yes.

"Can he cause multitudes, at one and the same time, to see, touch, and eat productions real in appearance, but ideal in reality ?—Yes.

"Can he have spirits, freed from matter, at his orders, and receive from them services ?—Yes.

"Can he bring about rain, winds, hail, or cause these natural phenomena to cease, at his will ?—Yes."†

Thus far M. Cahagnet, and happy it is for us that the magicians of our days know how to temper their power with a merciful kindness, and that they must be men of like sound-feeling as M. Lévi, else who could escape from mischief at their hands ? M. Lévi instructs us that he

"— Willingly gives lessons to serious individuals and teaches those who ask to be taught, but it is necessary that, in good faith, he should forewarn his readers that he does not inculcate fortune-telling or divination, neither does he make predictions, nor fabricate love-potions, nor lend himself to any sorcery or evocation. He is a man of science and not a juggler. He condemns energetically everything that religion reproves, and consequently he ought not to be confounded with those men who, without fear, advertise themselves and their science for dangerous and illicit uses."—(p. viii.)

But supposing for a moment that one or more of the magical confraternity would come forward and lend the world, without hesitation, his or their aid in doing some good or other for a short time. Surely so all-powerful a science ought to help somewhat more efficiently than it does our blundering efforts to leave the world around us a little better than we found it. But it is singular, that when these professors of the occult art speak of the amazing good flowing or which ought to flow from it, it is simply good in the *abstract*, and that the *concrete* examples which are paraded

* To drink *au chalumeau* is a little procedure of sorcery, by which we may quench our thirst or indulge a taste for vinous fluids at the expense of our neighbours. A straw or other convenient tube is rested, by one extremity, against the wall of a cellar, or dairy, or a hole is made in the wall. Certain words of power are next uttered, and then, at will, the wine in the cellar or the milk in the dairy may be subtracted through the pipe or the hole. It is pretended, M. Cahagnet remarks, that this subtraction is real, but he very properly doubts this. He can produce the same phenomenon, but it is an ideal one. He proceeds in the manner we have detailed, but "the wine (or the milk) which issues from his magnetic points, exists only for the sensitive subject in whom he has created this particular spiritual appreciation."—(*Op. cit.* p. 461.)

The termination of the drinking-scene in *Faust*, when Mephistopheles produces wine from gimlet-holes in the table, is a true drinking *au chalumeau*.

† *Magie Magnetique.* Par L. A. Cahagnet. Paris. 1854. Pp. 26-29.

in illustration of the power of magic, under whatever name found, are invariably of a useless and *evil* tendency.

And this reminds us of a question which we had well-nigh overlooked. Our author tells us that many readers believe that magic is the science of the prince of darkness. He avows candidly that he himself entertains no fear of Satan. " I fear only those who fear the devil," was a remark of Saint Theresa's. But we are cautioned that railleries on this subject would be much misplaced, and M. Lévi then proceeds to discuss the relation which exists between the devil and science, or rather to consider the former from a scientific point of view. He deals with the personality of Satan even less respectfully than St. Dunstan, for at one bold stroke he pounds Satan into an impalpable powder, and then blows this irrecoverably to the wind. " It is a lamentable truth that moral evil exists ; it reigns in certain minds, it is incarnated in certain men ; it is then personified, then demons come to light, and the most wicked of these demons is Satan. This is all that I wish you to admit, and this it will not be difficult for you to accord to me." (p. 11.)

" —— Every man
Conjures the fiend of hell into himself
When passion chokes or blinds him."
" —— All are devils to themselves ;
And every man his own great foe."

This is what science teaches us anent the fallen one—" the science unpolluted and most pure (*Vierge et mère*), the science of which Mary is the sweet and luminous image, was it not predestined to crush also the head of the ancient serpent ?"—(p. 11.)

It follows, therefore, that if magic ever tend to ill, it is from the vileness of the wielder of the art, not of the science itself.

It is high time that we began to learn how it comes to pass that the wondrous things we have set forth can be possible.

" How is all this possible ? Because there exists a mixed agent, a natural and divine agent, corporal and spiritual, an universal plastic intermedium (médiateur), a common receptacle of the vibrations of movement and of the images of form, a mind and a force that may, in some sort, be called the *imagination of nature*. By this force all nervous organisms communicate secretly together ; from it arise sympathy and antipathy ; from it come dreams ; by it are produced the phenomena of second-sight and of extra-natural vision. This universal agent of the works of nature, is the *od* of the Hebrews and Chevalier Reichenbach (just so : our readers will now more clearly perceive why, a few sentences back, we classed the imaginations of the animal magnetizer and the magician proper together, as belonging to one and the same category, although expressed in different terms), it is the astral light of the Martinists, and we prefer this last appellation as being the most explicit one.

"The existence and possible usage of this force form the great arcanum of practical magic. It is this force which constitutes the wand of the thaumaturgist and the clavicle of black magic.

"It was the serpent in Eden which transmitted to Eve the seductions of a fallen angel.

"The astral light magnetizes, heats, enlightens, electrifies, attracts, repulses, vivifies, destroys, coagulates, separates, breaks to pieces, collects together any and everything under the impression of powerful volitions.

"God created it on the first day when he said Fiat Lux!

"This explains already all the theory of prodigies and miracles. How, in reality, could the good and the wicked be able to compel nature to make manifest exceptional forces? How could there be divine miracles and diabolical miracles? How could a reprobate, wandering, loose spirit have more force in certain circumstances than a just one, so puissant in its simplicity and wisdom, if we were not to suppose an instrument of which all may make use, under certain conditions, some for the greatest good, others for the greatest evil?"—(p. 19.)

Ask us not to expound, dear reader, these sentences. Because, frankly to confess, we belong to those unlearned, who, to use an apt simile of Tristram Shandy, Esquire, are busied in getting down to the bottom of the well, where Truth keeps her little court, while M. Lévi belongs to those learned who, in their way, are as busy in pumping her up through the conduit of dialectic induction, and who *concern themselves not with facts, but with reason.*

There is, however, one application of this hypothesis of astral light, in M. Lévi's work, which we imagine will interest highly the present generation, and which will serve partly as an exposition of the method in which the force operates. To get to this portion of M. Lévi's book we have to pass over the greater bulk of it untouched, and to plunge at once into the latter days of the nineteenth century. It would not be just to the author to skip over thus much of his labours without some comment, and we may remark, that we know no history of magic which surpasses M. Lévi's historical exercitations, and few that equal them in interest. The arrangement is good, the illustrations apt and striking, and many curious mystical drawings are scattered through the book. It is written by a man who is enthusiastic in his subject, and his enthusiasm and confiding faith imbue the writing. To those who are interested in studying eccentric and abnormal developments of thought, we can promise much interest and no slight instruction if they take up M. Lévi's book.

The example to which we have just referred as an illustration of the mode of action of the astral light, is *Table-turning*. M.

Lévi assumes, without even manifesting the ghost of a doubt, that under the influence of human magnetization the heaviest masses can be raised from the ground, and promenaded in space. Nay, he speaks of articles of furniture delivering themselves at the same time as somnambulists, to frensied dances, and becoming fatigued, and even smashing their fragile members in the mad career, while the ladies suffered nothing, and were, indeed, all the better for the exercise.

But how can these things happen? Listen!

"—— Weight exists only by reason of the equilibrium of the two forces (attraction and repulsion) of the astral light; augment the action of one of them, and the other will at once yield. Now, if the nervous apparatus (system) aspires and inspires this light in rendering it positive or negative (it is a custom of these gentlemen to pilfer terms from the experimental sciences, and to stalk under their cover when practicable), according to the personal hyper-excitations of the subject, all inert bodies submitted to its action, and impregnated with its life will become lighter or heavier, according to the flux and reflux of the light which carries along with it in the new equilibrium of its movement porous bodies and bad conductors around a new living centre, in the same manner as the stars are balanced, and projected, gravitating around the sun."—(p. 494.)

What follows is really a most interesting addition to our knowledge of psychical abnormities, and the novel designation made use of by our author may, from its aptness, be legitimately admitted into scientific usage.

He continues:—

(*Mediomaniacs.*)

"This eccentric power of attraction or of projection always supposes a sickly state in the individual who is the subject of it. All *mediums* are eccentric and badly balanced beings; *mediomania* presupposes or occasions a succession of other nervous manias, fixed ideas, distempered desires, vicious erotomania, penchants to murder or suicide. Among persons thus affected, moral responsibility seems no longer to exist; they act wrong, not knowing what is right; they weep with piety in the church, and yet give themselves up to hideous bacchanal revels; they have one mode of explaining everything, to wit, they are possessed by the devil. What would you have? What do you require? They no longer live in themselves; they are animated by a mysterious being, it is he who acts, not themselves, and his name is Legion!

"The reiterated essays of a healthy individual to create the faculties of a *medium*, occasion fatigue or illness, and may derange the reason. This has happened to Victor Hennequin, formerly editor of the *Democratie Pacifique*, and a member, after 1848, of the National Assembly. He was a young advocate of fluent and facile speech, he did not lack either instruction or talent, but he was infatuated with the works of

Fourrier. Exiled after the 2nd December, he gave himself up during his idleness to experiments on table-turning. Presently he was attacked with *mediomania*, and believed himself to be the instrument of revelations of the soul of the earth. He published a book entitled *Sauvons le genre humaine*, which was a mélange of phalansterian remembrances and Christian reminiscences, a last flicker of the dying reason. He persisted in his experiments, and insanity triumphed. In a last work, of which the first volume has been alone published, Victor Hennequin represents God as an immense polyp fixed in the centre of the earth, with antennæ and tentacles twisted gimlet-fashion, which pierced hither and thither through his brain, and that of his wife Octavia. Soon after this Victor Hennequin died in a lunatic asylum, in consequence of an access of furious dementia.

"We have heard speak of a fashionable lady who yielded herself to conversations with the pretended spirits of her furniture, and who, being scandalized beyond measure by the unbecoming answers of her candlestick-stand (gueridon), went to Rome in order to submit the heretic piece of furniture to the holy seat. She carried with her the culpable article, and made an auto-da-fé of it in the capital of the Christian world. Better to burn the furniture there than become mad, and truly for this lady the danger was imminent.

"Let us not laugh at these things, children of a rational age or of serious men, as the Count de Mirville attributes to the devil the inexplicable phenomena of nature."—(pp. 494—6.)

We may laugh, however, at an account which M. Lévi gives, in all faith, of a certain Henri Delaage, who possesses the gift of ubiquity, and who at times exercises so beneficial an influence upon those who have the good fortune to be thrown into contact with him, that when influenza raged in the winter, a little while ago, he had but to present himself in a room where any of the unhappy sufferers from the disorder were present, to effect an immediate cure. The waft of his presence alone sufficed for this, but sad to relate, the disease which he drove from others, clung most revengefully to himself, and has never since left him. Would that we could conjure the presence of this healing man into our library at this moment, for now, while we write, the conduits of our nostrils are streaming, our eyes distil tears, our throat is raked by a teasing cough, while the innermost recesses of the brain are ever and anon pounded by horrific sternutations.

M. Lévi's description of *mediomania* and *mediomaniacs* is in truth so vivid and good that it may at once, justly, be transferred to our text-books of psychological medicine, and it is none the less valuable that it comes from an unprofessional source.

Hasten we now to seek a few specimens of the concentrated wisdom of *the* "lofty science."

Magic, it would seem, "is the absolute science of equilibrium." It is, however, an essentially religious science, and it "presided over the formation of the dogmas of the ancient world, and"—the conclusion of the sentence may be commended to Mr. Buckle's notice, as calculated to facilitate his labours—"has been thus the foster-mother of all civilizations. Mother, chaste and mysterious, who, whilst suckling with poesy and inspiration nascent generations, covers her face and her breast!"

Before everything, the "lofty science" teaches us to believe in God, and to adore him without seeking to define him, because it often happens that with us (a consequence of our imperfection) a defined God is in some degree a finite God.

After God, it gives us to know the sovereign principles of things, the eternal mathematics, and the balanced forces. "It is written in the Bible that God has disposed everything by weight, number, and measure. Here is the text: *Omnia in pondera et numere et mensura disposuit Deus*. Thus, weight, that is to say equilibrium, number, quantity, and measure, that is to say proportion: such are the eternal or divine foundations of the science of nature. The formula of equilibrium is this: "*Harmony results from the analogy of contraries.*"

Then, we hear, that the letters of the sacred alphabet, as expounded by the ancient hierophants, contain potentially all the secrets of nature. By sundry arrangements of this alphabet, every possible combination of natural forms can be discovered. Further, we are told, that God, as it is recorded in Genesis, made man in his own image. "Now man being the living *resumé* of creation, it follows that creation also is in the image of God. There are in the universe three things: *spirit*, the *plastic intermedium*, and *matter*." The ancients rightly symbolized these by *sulphur*, *mercury*, and *salt*. But the three constituents of the Universe unite in one, *light;* "Light, positive or igneous, the volatile sulphur; light, negative or rendered visible by the vibration of fire, the fluid ethereal mercury; and light neutralized, or shade, mixed, coagulated, or fixed under the form of earth or salt."—(p. 531.)

This must suffice; for it cannot be but that this bathos of wisdom is as painful to read as tedious to write.

It remains to us now but to illustrate the certitude of magical science as an instrument of philosophical and religious inquiry. We have already said that it renders "reason as infallible as mathematics."

Towards the close of his book, M. Lévi proposes and boldly grapples with the following questions:—

1. Can we escape death?

2. Does the philosopher's stone exist, and in what manner can we find it?

3. Can we cause spirits to serve us?

4. What is the clavicle, ring, and seal of Solomon?

5. Can we foretell the future with certainty?

6. Can we cause good or evil at will by magical influence?
7. What is needful to constitute a true magician?
8. In what do the forces of black magic precisely consist?

Of course we can escape death, and in two manners, "in time and in eternity. In time by *curing* all maladies and evading the infirmities of old age: in eternity, in perpetuating the memory of our personal identity through the transformations of existence." The principles upon which these conclusions are framed, are summed up, but we naturally hasten on to seek at least in what fashion we can effect the escape from death in time; but as may be imagined, no such information is forthcoming. We are overwhelmed instead beneath avalanches of abstractions. We are further told, that the philosopher's stone most certainly exists "*in theory*," and that if we would find it (it has not yet been found, by the way,) "it is indispensable that we should seek it, at least it will not be found by chance. We (M. Lévi) have said sufficient to facilitate and direct researches."

And yet these responses are about the clearest given, but they by no means correspond with the magnificent promises with which we started. Those who wish to know anything of Solomon's clavicle, ring, and seal, had much better turn to Lane's edition of the *Arabian Nights*, and read the tales in which the said occult articles figure. One portion of the answer to the eighth question must, indeed, be quoted. It is an unique specimen of Gallic blasphemy:—

"Between Jesus Christ and Napoleon, the world of the marvellous remained void. Napoleon, the Word of war, this armed Messiah, came fatally, without knowing it, to complete the Christian world. The Christian revelation taught us but to die; the Napoleonic civilization ought to teach us to vanquish. Of these two and apparently contradictory words, devotion and victory, to suffer, to die, to combat, to conquer, is formed the great arcanum, HONOUR!"

Could we desire a better illustration of *chronic national mania*?

We might, perhaps, with propriety lay down our pen here. We have, we think, satisfactorily established the proposition with which we started, that our modern magicians are by no means inferior (at least in professions) to those of an earlier date. We have also achieved what we intended—the contribution of a waif of information on the psychical eccentricities, or abnormities, if you will, of the present period. We cannot, however, resist the temptation to tag a moral to our recital.

It can need only a hint to our readers (if the thought has not been painfully present to them during the whole of their reading) to direct their attention, and to lead them at once to apprehend that the principles and practices taught by M. Lévi are but a phase of that pseudo-scientific delusion, animal magnetism,* which has settled into a chronic state among us, as well as of the epidemic delusions of table-turning and spirit-rapping which have so recently prevailed in this country. We are too apt to forget that the cessation of the two latter delusions as *epidemics* by no means implies their *total* cessation. It would be easy to prove, if it were needful, that both delusions, as well as that of animal magnetism, exist in a chronic form. Now, it must be evident to those who have carefully watched the progress of the delusions in question, and who have noted (as we may observe, for one example, in M. Lévi's book) the manner in which the believers in any one of the delusions named seize upon, as confirmatory of their peculiar form of belief, whatever turns up in any other form of a congenerous character, that the condition of mind which leads the possessor so greatly astray, must be very similar in every instance. Further, it will also be comprehended that the determination to this or that peculiar form of delusion depends, most probably, upon the point and the circumstances from which the believer started on his quest into the marvellous.

We assume that the substratum of these wide-spread delusions and their congeners chiefly results from a fundamental error of education, both of the emotions and intellect, *in early life*, and that this substratum and the causes engendering it are pretty much the same in every case. We are not going to dogmatize on the precise nature either of the substratum or its causes. These cannot be dealt with at the fag-end of an article, and they have often been treated by abler pens than ours. Our object is bounded here to impressing on the minds of those who may glance their eye over these words, that the recrudescence of delusions such as we have specified, and their chronic character, decisively indicate that the sources from which they are developed are both wide-spread and but little inferior in intensity, although, we have little doubt, less injurious in character and less extended in their operation, than the superstitious delusions of the middle ages.

If we would endeavour earnestly to break up the delusional substratum of which we have spoken, this, we aver, is to be done during the interval of the epidemic outbreaks, and not at the time of their occurrence. Then the mischief has been done. We cry out loudly, Wolf! wolf! but before help arrives, the wolf has devoured many a hapless lamb, which, while the sun shone brightly, and the birds chirped loudly, and the leaves trembled

* It would be more correct to say that animal magnetism is but a phase of magic, the latter being of more ancient date.

into additional life in the cheering breeze, we suffered to dally with itself in idle play,* while that we reposed upon the green-sward, haply dreaming of Arcadia. We hold, therefore, that it is an important duty to watch during times of repose from delusions, their ordinary every-day course, and to ascertain their actual standing. By so doing we always hold in sight the deleterious mental poison, and not only so, but by having its natural development before us, we run less danger of under-estimating those slight indications of its presence which form a sure sign of predisposition to its epidemic influence. Each individual who has escaped the insidious poison—how insidious, we have surely sufficiently seen in the book we have just analysed, in which we behold one in holy orders tainting with vain imaginations Gospel truths to their very source—each individual, we say, who has escaped from the infection may thus be tempted to inquire for himself in what the predisposition to be affected by these modern superstitions consists. If he do so, he will doubtless learn much, and, perhaps, do more to prevent its development, as well in himself as in those around him, than if a shower of right precepts and happy truisms were cast at him.

If it be said that this our didactic method may be fittingly compared to a guide-post, the inscriptions on which have been partially effaced, and can only be deciphered with difficulty, we admit the charge implied, and standing reproved, we will throw into our teaching a postscript which, in a concrete form, pierces pretty nigh to the root of the whole matter.

The sagacious father of Tristram Shandy, Esquire, has expressed an opinion that "there is a north-west passage to the intellectual world." A somewhat similar opinion, expressed or understood, exists very widely among men as to the world of natural science, and they seek to carry it to fruition much in the same way as the aforementioned gentleman thought was practicable in regard to the intellectual world—to wit, by the aid of the *auxiliary verbs.* "The use of *auxiliaries,*" he asserts, "is at once to set the soul agoing by herself upon the materials as they are brought to her, and by the versatility of the great engine round which they are twisted, to open new tracts of inquiry, and make every idea engender millions." The wisdom of this opinion is profound, but, as Bacon would have said,† Mr. Shandy, senior, while admiring and extolling the powers of the human mind, *did not search for its real helps.* Thus the man who has learned to know the value of the auxiliaries, without at the same time be-

* "——— Poor hapless ones
That leave their mother's milk
To dally with themselves in idle play."—*Dante.*

† Novum Organum, Aph. 9.

coming acquainted with the errors that beset their use, is apt, as Mr. Shandy would have put it, to adopt a form of ratiocination on any given subject, somewhat like this:—*Is it so? Was it so? Will it be so? Could it be so? Would it be so? May it be so? Might it be so?*—and the imagination gaining strength with this capital exercise, soon adopts the affirmative formulæ, *It is so: It was so: It ought to be so: It must be so:* It SHALL be so. If the inquirer have early learned, however, that these auxiliary forms have their peculiar pitfalls and stumbling-blocks, he will not be any the less an inquirer, but he will learn to look modestly upon his own powers, and having this modesty he will be placed in a more favourable position for escaping the rampant pseudo-scientific follies which haunt the world.

---

